# The topography of infratentorial lesions in depression and anxiety in multiple sclerosis

**DOI:** 10.1007/s00415-025-13167-0

**Published:** 2025-05-26

**Authors:** Federica Giofrè, Alessandra Lugaresi, Flavia Baccari, Elaine Lui, Stefanie Roberts, Charles Malpas, Tomas Kalincik

**Affiliations:** 1 Dipartimento di Scienze Mediche e Chirurgiche, Alma Mater Studiorum Università di Bologna, Bologna, Italy; 2 UOSI Riabilitazione Sclerosi Multipla, IRCCS Istituto delle Scienze Neurologiche di Bologna, Bologna, Italy; 3 Dipartimento di Scienze Biomediche e Neuromotorie, Alma Mater Studiorum Università di Bologna, Bologna, Italy; 4 Unità Operativa di Epidemiologia e Statistica, IRCCS Istituto delle Scienze Neurologiche di Bologna, Bologna, Italy; 5 Department of Radiology, The University of Melbourne, Melbourne, Australia; 6 Department of Medical Imaging, The Royal Melbourne Hospital, Melbourne, Australia; 7 CORe, Department of Medicine, The University of Melbourne, Melbourne, Australia; 8 Department of Neurology, Neuroimmunology Centre, The Royal Melbourne Hospital, Melbourne, Australia

**Keywords:** Demyelination, Raphe nucleus, Locus coeruleus, Posterior cerebellum, Depression, Anxiety

## Abstract

**Background:**

Depression and anxiety are highly prevalent in multiple sclerosis and significantly impact patient outcomes. However, their link to specific areas of demyelination remains unclear.

**Objectives:**

This study examines the association between infratentorial lesions and depression or anxiety, focusing on three regions of interest: raphe nuclei, locus coeruleus, and cerebellar lobule VIIA.

**Methods:**

Patients were recruited from the cognitive neuroimmunology clinic at the Royal Melbourne Hospital. Participants were categorised as belonging to the groups ‘depression’/‘no depression’ and ‘anxiety’/‘no anxiety’ based on SPECTRA Indices of Psychopathology. MRI scans were examined for lesion presence in regions of interest. Association analyses were performed using multivariable logistic regression, adjusting for demographics, clinical parameters, and therapy.

**Results:**

Of 73 patients, 21 (29%) had clinically relevant depressive symptoms, and 18 (25%) had anxiety. Depression was significantly associated with lesions in the raphe nuclei (47.6% vs. 17.3%, OR 10.5, 95%CI 1.9–57.5, *p*=0.007) and locus coeruleus (38.1% vs. 15.4%, OR 20.5, 95%CI 2.3–184, *p*=0.007). Anxiety showed a potential association with locus coeruleus lesions (38.9% vs. 16.4%, OR 8.62, 95%CI 0.9–79.2, *p*=0.057).

**Conclusions:**

Depression in multiple sclerosis is associated with lesions within serotoninergic and noradrenergic brainstem nuclei. No definitive anatomical substrate for anxiety was identified. These findings suggest that inflammatory structural changes may underlie mood disorders in multiple sclerosis, potentially serving as early imaging markers of susceptibility to depression.

## Introduction

Multiple sclerosis (MS) is the most common demyelinating disease of the central nervous system in young adults [1]. A variety of comorbid conditions can associate with MS. Among these, affective disorders are common, presenting a higher frequency in MS than in the general population [2]. Depression and anxiety are the most prevalent affective disorders in people with MS, sharing common risk factors and often overlapping presentations. Depression and anxiety often occur simultaneously. [3] Depression affects around 30% of people with MS, which exceeds the expected prevalence 3–10 times [4, 5]. Anxiety is reported in 36% of people with MS compared to 30% of the general population [6].

In MS, mood disorders are associated with lower quality of life, impaired cognitive performance, fatigue, lower treatment adherence, increased tendency to substance abuse, and higher suicide risk [3, 5, 7–9]. Understanding the pathogenesis of these disorders in MS is important for a more effective diagnosis, treatment, and prevention. The origins of mood disturbances in MS are believed to be multifactorial, including a reactive component and, possibly, a unique neuropathological mechanism [3]. Primary depression and anxiety are associated with dysfunctions of the adrenergic and serotoninergic systems within the brain. These systems extend into many cortical and subcortical networks and are closely involved in the control of mood. [10] A limited number of neuroimaging studies in MS-related affective disorders published to date have focused on supratentorial findings, showing associations between depression and T2 hyperintense lesions in frontal, temporal, and parietal lobes, hippocampus, amygdala, cingulate cortex, fusiform gyrus, and para-hippocampal region [3, 5, 7–9, 11]. Findings of altered functional connectivity between raphe nuclei (RN) and subcortical nuclei in people with MS and depression has led to the hypothesis that MS may increase the risk of depression through disruption of serotonergic networks [11, 12]. The potential associations between MS-related structural changes of the brain and anxiety are even less understood [5].

Here, we studied the potential relationships between infratentorial demyelinating lesions and depression or anxiety in MS, using a prospective cohort assessed in a specialised cognitive clinic. Given the known differences in the anatomical substrates of depression and anxiety, both conditions were included and studied in separate models. We focused on the RN, locus coeruleus (LC), and cerebellar lobule VIIA. We hypothesised that the prevalence of clinically significant depressive and anxiety symptoms is higher in people with MS lesions in these regions of interest.

## Methods

### Participants

This study was approved by the Royal Melbourne Hospital Human Research Ethics Committee. The screened patients were assessed in the Cognitive Neuroimmunology clinic of the Neuroimmunology Centre based on specialist referrals from Neuroimmunology clinics, typically on the basis of a cognitive complaint or an administrative need of formal cognitive assessment. Patients were included based on the following criteria: (1) definite diagnosis of MS [13], (2) completion of the SPECTRA Indices of Psychopathology [14], and (3) availability of a brain MRI scan with demyelination protocol completed within one year of the SPECTRA questionnaire. Patients were included irrespective of their disease-modifying and antidepressant/anxiolytic therapy.

### Demographic and clinical data

The following demographic and clinical data were recorded within one year of completing SPECTRA: patient age; sex; MS phenotype; the Expanded Disability Status Scale (EDSS) score recorded by Neurostatus-certified raters [15]; and disease-modifying treatment at the time of the SPECTRA assessment. We also collected data on previous interactions with a psychologist or psychiatrist, and the number and type of psychotropic medications the patients were treated with at the time of completing SPECTRA. Cognitive performance was screened using the validated Brief International Cognitive Assessment for MS (BICAMS) [16], consisting of the Symbol Digit Modality Test (SDMT) [17], the second edition of the California Verbal Learning Test [18], and the revised Brief Visuospatial Memory Test [19]. Standardised SDMT percentiles were used as an adjustment variable in the analyses of the relationship between lesion topography and depression/anxiety.

### Assessment of depression and anxiety

Symptoms of depression and anxiety were assessed using the 96-item self-administered SPECTRA questionnaire [14]. This questionnaire measures the symptomatic expression of different psychiatric disorders and organises them into higher-order spectra and subspectra clusters, following the hierarchical-dimensional model of psychopathology and multivariate research. Each SPECTRA T score can be interpreted either dimensionally, to represent a patient’s standing on a psychological state or trait scale, or using cut scores [20]. A T score higher than or equal to 70 was considered clinically elevated.

### MRI imaging and evaluation

MRI of the brain was acquired using a 3-Tesla MRI scanner (Skyra, Siemens, Erlangen, Germany). The brain demyelination MRI protocol included 3D T1-Weighted Imaging (3D-T1 WI MPRAGE, 1 mm isotropic resolution, repetition time (TR)=2300 ms; echo time (TE)=2.98 ms; inversion time (TI)=900 ms; flip angle (FA)=9°; acquisition time (TA)=5:09 min), 3D Fluid Attenuated Inversion Recovery (3D-FLAIR) imaging (1 mm isotropic resolution, TR=5000 ms; TE=408 ms; TI=1800 ms; FA=120°; TA=3:50 min), axial T2-Weighted Imaging (T2 WI, 0.5 x 0.5 x 3 mm resolution, 0.9 mm gap, TR=7880 ms; TE=105 ms; FA=150°; TA=01:26 min), and Diffusion-Weighted Imaging (1 x 1 x 4 mm resolution, TR=8000 ms; TE=64 ms; FA=180°; TA=3:38 min). Morphometry measures were obtained using MorphoBox (Syngovia VB30B.36, Siemens, Erlangen, Germany) [21].

### Lesion assessment and anatomical landmarks of regions of interest

**Fig. 1:**
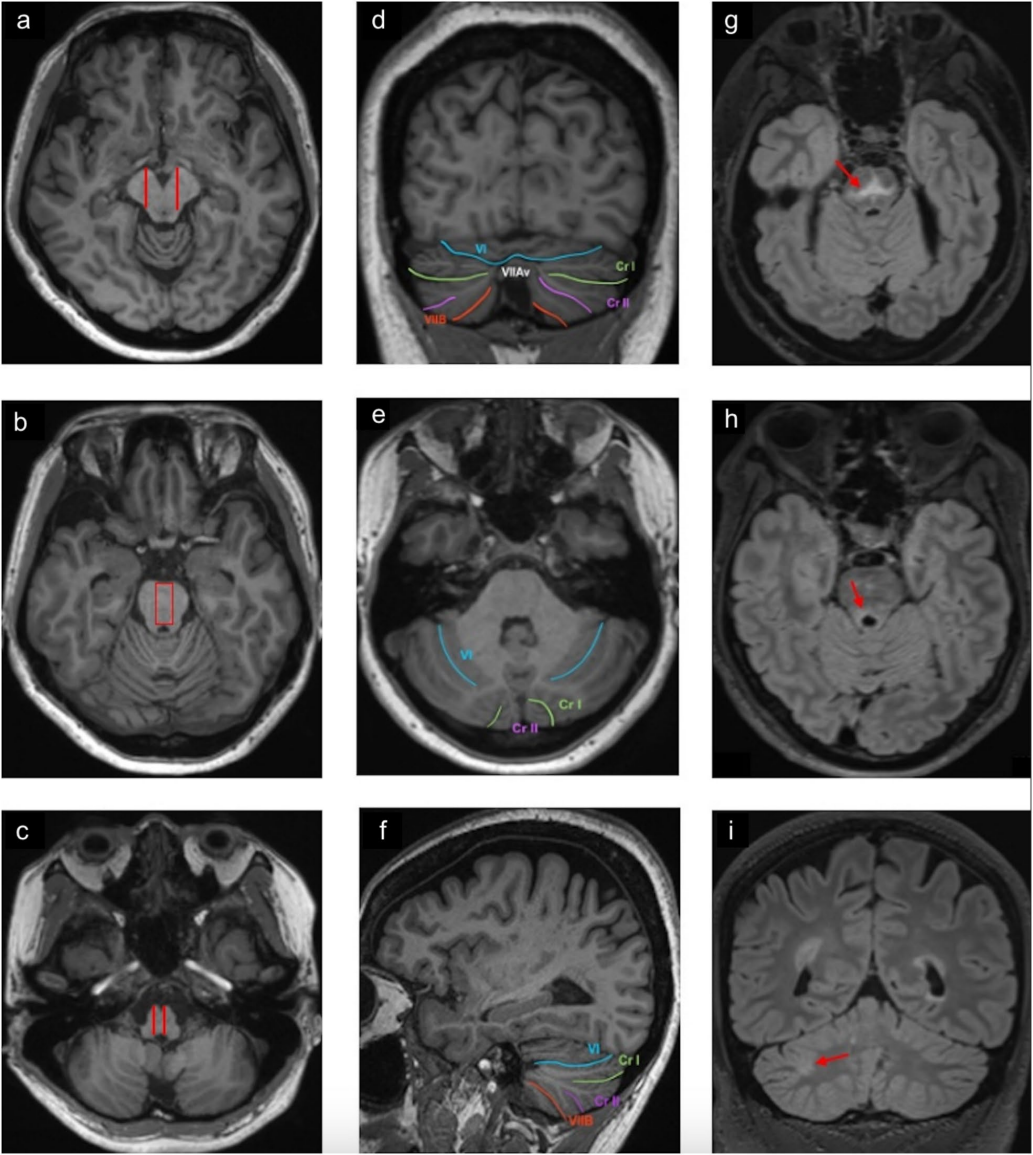
Regions of interest. The examples demonstrate our delineation of medial and lateral midbrain (**a**), pons (**b**) and medulla oblongata (**c**). Cerebellar lobule VIIA: crura (Cr I and Cr II) and vermis (VIIAv) are shown in panels (**d**), (**e**) and (**f**). (**g**), (**h**) and (**i**) show examples of T2 hyperintense demyelinating lesions (arrows) involving the raphe nuclei (**g**) locus coeruleus (**h**) and cerebellar lobule VIIA (**i**)

We defined a lesion as a discrete hyperintensity on T2-weighted 3D-FLAIR imaging that is visualised on at least 2 planes or 2 contiguous slices in the same plane after excluding for artefact. For lesion load assessment, lesions were documented if they were located within the brainstem or cerebellum according to the following regions of interest defined on the 3D-T1-weighted images: *Medial and lateral midbrain* were delineated by two antero-posterior lines drawn through the most anterior part of the cerebral peduncles (Fig. 1a). *Medial and lateral pons* were delineated with the method used by Supprian and colleagues [22] where each axial slice of pons was marked with a square delimited anteriorly by the pontine cistern and posteriorly by the cerebral aqueduct, which includes the RN complex (Fig. 1b). The area of the square was kept constant across all slices but was adjusted at each slice for the location of the median RN. *Medial and lateral medulla oblongata* were delineated antero-posteriorly using the position of the pyramidal tracts (Fig. 1c). The lesions were counted manually within each of the above regions as well as left and right cerebellar hemispheres, and cerebellar vermis. This was performed by FG and reviewed by a senior radiologist (EL).

For lesions involving RN, we used the anatomical descriptions of Hornug [23]: lesions localising to the midline tegmentum along the rostro-caudal axis of the brainstem (Fig. 1g). For lesion involving the LC, we used the *in vivo* definition of Fernandes et al. [24]: lesions in the posterior rostral pons, localised approximately 1 mm caudal the IV ventricle, 3 mm lateral to the midline, and 14–21 mm rostral to the pontomedullary junction (Fig. 1h). For lesions involving cerebellar lobule VIIA (i.e. crus I; crus II; vermal lobule), we localised them according to the description by Schmahmann et al. [25] (Fig. 1d-f). For example, a lesion between the superior posterior fissure and the ansoparamedian fissure was considered to involve VIIA (Fig. 1i).

### Statistical analyses

Data are presented using mean and standard deviation (SD) or count and percentage, as appropriate. Standardised mean difference (SMD) was used to describe unadjusted differences between the groups. The normality of continuous variables was evaluated visually and using Shapiro–Wilk and Kolmogorov–Smirnov tests.

Multivariable logistic regression models were used to investigate the associations of radiological parameters with the outcomes of interest (depression and anxiety), adjusted for demographics, clinical characteristics, and therapy. Variables included as potential confounders were selected a priori, based on a directed acyclic graph, and were further refined based on a correlation matrix to exclude strongly collinear variables. A separate model was used for all combinations of depression or anxiety with lesioned RN, LC, and cerebellar lobule VIIA. A distinct analysis was conducted for the relationship between depression/anxiety symptoms and lesions in the nine infratentorial regions of interests (i.e. medial and lateral midbrain; medial and lateral pons; medial and lateral medulla; right cerebellar hemisphere; left cerebellar hemisphere; cerebellar vermis). Benjamini–Hochberg correction was applied to correct for false discovery rate in multiple hypothesis testing. Statistical analyses were performed using Stata version 16.

## Results

73 patients were included and followed in this study from May 2019 to August 2022. Data were retrieved in December 2022. Demographic, clinical, and radiological characteristics of interest are summarised in Table 1. The study sample was representative of the general MS population, with mean age of 41.3 years, 75.3% females, mean EDSS of 2.2 steps, and 68 patients (93.2%) diagnosed with relapsing-remitting MS. 54.8% used any psychotropic therapy, including 19.2% of patients treated with antidepressants, 9.6% treated with anxiolytics, and 26% treated with therapies with combined effects. 19 patients (26%) had lesions involving the RN, 16 (21.9%) involving the LC, and 16 (21.9%) involving cerebellar lobule VIIA.

**Table 1:** Characteristics of the studied cohort

	Count or mean	% or standard deviation
Total participants	73	
Age, y, mean	41.3	11.4
Disability, EDSS step^a^, mean	2.2	1.8
Sex, n		
Female	55	75.3
Male	18	24.7
MS phenotype, *n*		
Relapsing-remitting MS	68	93.2
Primary progressive MS	4	5.5
Secondary progressive MS	1	1.4
Disease-modifying therapy, *n*		
No/Low-/Moderate-efficacy therapy	13	17.8
High-efficacy therapy	60	82.2
Psychotropic medication, *n*		
No therapy	33	45.2
Antidepressants	14	19.2
Anxiolytics	7	9.6
Combined therapy	19	26
Number of psychotropic medications, *n*		
0	33	45.2
1	24	32.9
2+	16	21.9
Previous engagement with psychology/psychiatry^b^, *n*		
Yes	41	57.7
SDMT score^c^, standardised percentile, mean	− 0.6	1.1
Lesions within regions of interest, n		
Raphe nuclei, *n*	19	26
Locus coeruleus, *n*	16	21.9
Cerebellar lobule VIIA, *n*	16	21.9
Normalised brain volume, %, mean	77.5	3.9

Low-/Moderate-efficacy disease-modifying therapy includes glatiramer acetate, teriflunomide, dimethyl fumarate; High-efficacy disease-modifying therapy includes alemtuzumab, ocrelizumab, natalizumab, fingolimod, cladribine; Antidepressant therapy includes amitriptyline, citalopram, fluoxetine, nortriptyline, mirtazapine, sertraline, vortioxetine, venlafaxine, desvenlafaxine, agomelatine; Anxiolytic therapy includes clonazepam, oxazepam, temazepam, pregabalin; Combined psychotropic therapy includes duloxetine, escitalopram, paroxetine

*EDSS, Extended Disability Status Scale; MS, multiple sclerosis; SDMT, Symbol Digit Modalities Test*

^*a*^*missing n=4*

^*b*^*missing n=2*

^*c*^*missing n=5*

### Depression

Of the 73 included patients, 21 (29%) were experiencing clinically relevant symptoms of depression. The demographic and clinical characteristics were similar between patients with and without depression (Table 2).

**Table 2:** Characteristics of patients stratified by their depression and anxiety status

	No depression		Depression			No anxiety		Anxiety		
	Count or mean	% or SD	Count or mean	% or SD	SMD	Count or mean	% or SD	Count or mean	% or SD	SMD
Patients, *n*	52	71	21	29		55	75	18	25	
Age, y, mean	42	11.2	39.6	11.8	0.21	42	11.6	39.1	10.7	0.27
Disability, EDSS step, mean	2.1	1.8	2.5	1.9	− 0.24	2.1	1.7	2.6	2.1	− 0.31
Sex, *n*										
Female	38	73.1	17	81	− 0.18	40	70.7	15	83.3	− 0.25
Male	14	26.9	4	19		15	27.3	3	16.7	
MS phenotype, *n*										
Relapsing-remitting MS	49	94.2	19	90.5	− 0.18	53	96.4	15	83.3	− 0.48
Primary progressive MS	2	3.9	2	9.5		1	1.8	3	16.7	
Secondary progressive MS	1	1.9	0	0		1	1.8	0	0	
Disease-modifying therapy, *n*										
No/Low-/Moderate-efficacy therapy	11	21.2	2	9.5	− 0.32	10	18.2	3	16.7	− 0.04
High-efficacy therapy	41	78.8	19	90.5		45	81.8	15	83.3	
Psychotropic medication, *n*										
No therapy	26	50	7	33.3	− 0.45	26	42.3	7	38.9	
Antidepressants	10	19.2	4	19		10	18.2	4	22.2	− 0.17
Anxiolytics	6	11.6	1	4.8		6	10.9	1	5.6	
Combined therapy	10	19.2	9	42.9		13	23.6	6	33.3	
Number of psychotropic medications, *n*										
0	26	50	7	33.3	− 0.35	26	42.3	7	38.9	− 0.32
1	16	30.8	8	38.1		19	34.5	5	27.8	
2+	10	19.2	6	28.6		10	18.2	6	33.3	
Previous engagement with psychology/psychiatry, *n*										
Yes	27	52.9	14	70	− 0.35	28	51.8	13	76.5	− 0.52
SDMT score, standard percentile, mean	− 0.5	1	− 1	1.2	0.40	− 0.5	1	− 1.1	1.1	0.59
Lesions within regions of interest, *n*										
Raphe nuclei, n	9	17.3	10	47.6	− 0.67	12	21.8	7	38.9	− 0.37
Locus coeruleus, n	8	15.4	8	38.1	− 0.52	9	16.4	7	38.9	− 0.51
Cerebellar lobule VIIA, n	12	23.1	4	19	0.01	12	21.8	4	22.2	− 0.01
Normalised brain volume, %, mean	77.2	3.8	78.2	3.9	− 0.26	77	3.9	79	3.5	− 0.54

*SD* standard deviation, *SMD* standardised mean difference

**Fig. 2:**
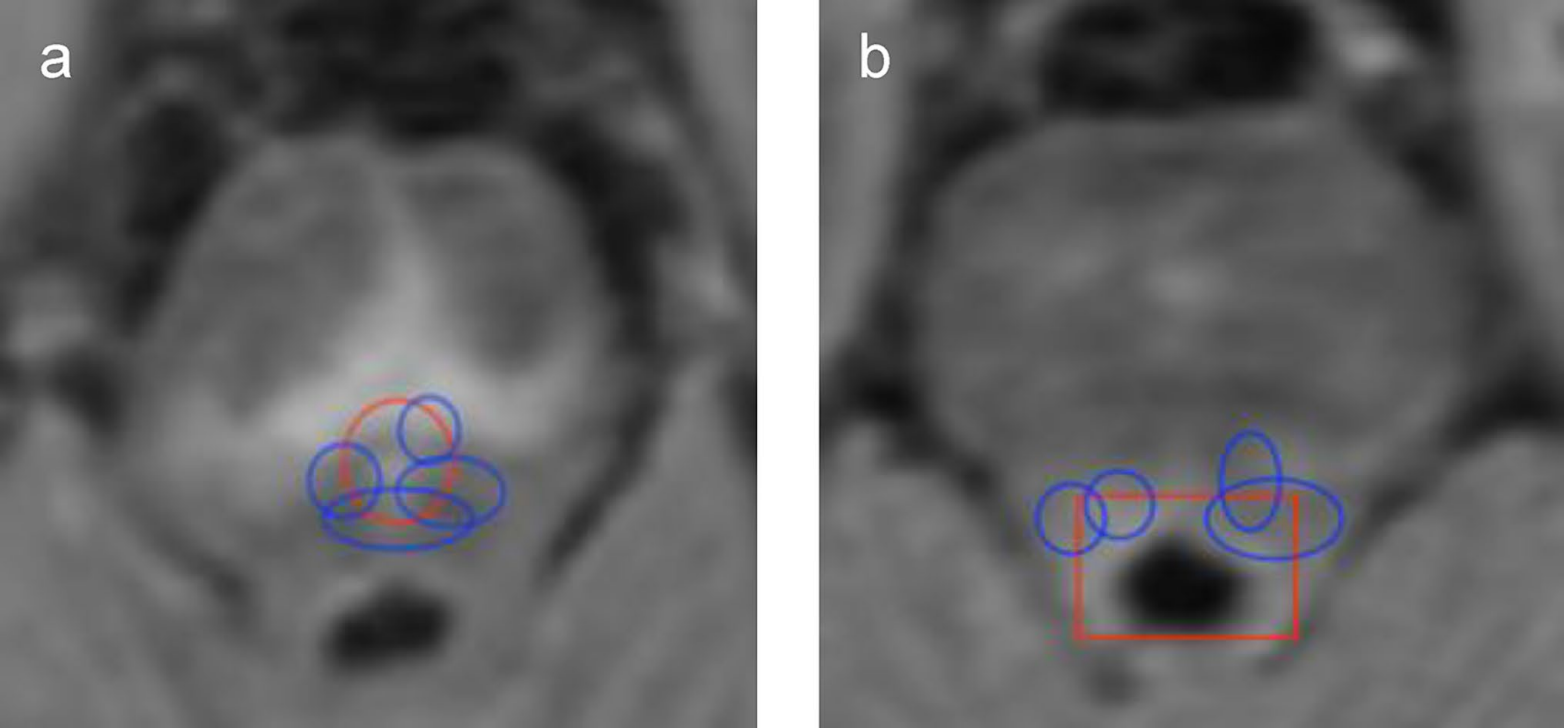
Lesion topography in MS patients with depression. **a** Overlayed on the lesion example in Fig.1g, the red circle delineates the serotoninergic RN located in the rostral pons within the ventral periaqueductal gray and the blue circles represent lesions involving the RN in our cohort. **b**. Overlayed on the lesion example in Fig.1h, the red square depicts the noradrenergic LC located in the dorsal pons below the floor of the IV ventricle and the blue circles represent examples of lesions within the LC.

Among patients with depressive symptoms, T2 hyperintense lesions more commonly involved RN (47.6% vs. 17.3%, SMD=− 0.67) and LC (38.1% vs. 15.4%, SMD=− 0.52) compared to those who did not fulfil the SPECTRA definition of depression. The multivariable models confirmed this association of depression with lesions involving RN (OR 10.5, 95%CI 1.9–57.5, *p*=0.007; Fig. 2a) and LC (OR 20.53, 95%CI 2.3–184, *p*=0.007; Fig. 2b) after adjusting for age, sex, EDSS step, disease-modifying therapy, SDMT score, previous engagement with psychology/psychiatry, use of psychotropic medications, and brain volume (Table 3). The analysis found no evidence for association between depression and lesions involving cerebellar lobule VIIA (OR 0.72, 95%CI 0.2–3.4, *p*=0.7).

**Table 3:** Associations of lesioned raphe nuclei, locus coeruleus, and cerebellar lobule VIIA with depression

	RN model			LC model			Cerebellar lobule VIIA model		
	OR	95%CI	p-value	OR	95%CI	*p*-value	OR	95%CI	*p*-value
Lesioned regions of interest (RN, LC, cerebellar lobule VIIA)	10.5	1.92–57.47	0.007	20.53	2.29–184.02	0.007	0.72	0.15–3.39	0.676
Age, y	1.04	0.95–1.13	0.400	0.98	0.90–1.07	0.640	1.01	0.93–1.08	0.898
Sex, female	2.71	0.52–14.1	0.237	5.26	0.82–33.67	0.080	2.32	0.49–11.02	0.289
Disability, EDSS step	1	0.66–1.52	0.997	1.15	0.78–1.7	0.476	1.10	0.77–1.58	0.596
Disease-modifying therapy, high-efficacy therapy	4.51	0.42–48.25	0.213	1.32	0.17–10.26	0.790	3.71	0.52–26.54	0.192
SDMT score	0.73	0.33–1.62	0.442	0.81	0.39–1.68	0.571	0.58	0.29–1.16	0.121
Previous engagement with psychology/ psychiatry	2.53	0.59–10.89	0.211	1.56	0.39–6.31	0.533	1.71	0.48–6.13	0.407
Psychotropic medication	3.75	0.84–16.7	0.083	7.78	1.17–51.77	0.034	2.41	0.66–8.75	0.182
Normalised brain volume, %	1.16	0.91–1.47	0.223	1.12	0.91–1.38	0.294	1.06	0.87–1.29	0.554

The table presents results of three multivariable models.

*95%CI* 95% confidence interval, *LC* locus coeruleus, *OR* odds ratio, *RN* raphe nuclei

Hyperintense T2 lesions were more common in the medial midbrain (OR 10.4, 95%CI 1.1–101.8, *p*=0.044) and the medial pons (OR 8.3, 95%CI 1.5–45.6, *p*=0.015) among patients with depressive symptoms in comparison to those without them (Table 5).

### Anxiety

Based on the SPECTRA questionnaire, 18 of 73 patients (25%) were experiencing clinically significant symptoms of anxiety. The group with anxiety appeared to be enriched with patients with primary progressive MS (16.7% vs. 1.8%, SMD=− 0.48), patients with lower standardised SDMT score (− 1.1 vs. − 0.5, SMD=0.59), and patients previously in care of psychologist or psychiatrist (76.5% vs. 51.8%, SMD=− 0.52) (Table 2).

T2 lesions tended to more commonly involve the LC in the patients with than those without anxiety symptoms (38.9% vs. 16.4%, SMD=− 0.51). However, this observation did not reach the required level of evidence in the fully adjusted model (OR 8.62; 95%0.9–79.2; *p*=0.057) (Table 4). We did not find any evidence of a difference between the patients with and without anxiety in the involvement of RN, VIIA, and the regions of interest within the brainstem/cerebellum (Tables 2, 4, 5).

**Table 4:** Associations of lesioned raphe nuclei, locus coeruleus, and cerebellar lobule VIIA with anxiety

	RN model			LC model			Cerebellar lobule VIIA model		
	OR	95%CI	*p*-value	OR	95%CI	p-value	OR	95%CI	p-value
Lesioned regions of interest (RN, LC, cerebellar lobule VIIA)	3.04	0.52–17.85	0.219	8.62	0.94–79.16	0.057	1.04	0.17–6.34	0.964
Age, y	1.04	0.94–1.14	0.479	1	0.91–1.11	0.990	1.02	0.93–1.12	0.708
Sex, female	7.76	0.97–62.35	0.054	12.14	1.37–107.62	0.025	7.28	0.96–55.15	0.055
Disability, EDSS step	1.23	0.77–1.95	0.383	1.35	0.85–2.12	0.201	1.29	0.83–2.01	0.267
Disease-modifying therapy, high-efficacy therapy	5.79	0.33–100.67	0.228	2.26	0.15–34.47	0.559	4.67	0.31–69.47	0.263
SDMT score	0.33	0.12–0.92	0.035	0.41	0.15–1.1	0.078	0.32	0.12–0.85	0.021
Previous engagement with psychology/ psychiatry	3.9	0.76–20.08	0.103	3.47	0.64–18.87	0.150	3.4	0.69–16.74	0.132
Psychotropic medication	1.67	0.36–7.72	0.514	2.97	0.48–18.28	0.240	1.4	0.32–6.08	0.656
Normalised brain volume, %	1.41	1.05–1.88	0.020	1.41	1.06–1.86	0.017	1.35	1.03–1.76	0.032

*The table presents results of three multivariable models.*

*95%CI, 95% confidence interval; LC, locus coeruleus; OR, odds ratio; RN, raphe nuclei*

**Table 5:** Associations between the topographic location of hyperintense T2 lesions and depression or anxiety

	Total (*n* = 73)	%	Depression			Anxiety		
			OR	95%CI	*p*-value	OR	95%CI	*p*-value
Medial midbrain 0 1+	71 2	97.2 2.8	10.4	1.06–101.82	0.044	3.44	0.09–137.6	0.512
Lateral midbrain 0 1+	64 9	87.7 12.2	2.4	0.48–12	0.288	22	0.91–532.4	0.057
Medial pons 0 1+	51 22	69.9 30.1	8.3	1.52–45.6	0.015	2.52	0.42–14.98	0.309
Lateral pons 0 1+	47 26	64.4 35.6	1.04	0.26–4.07	0.959	4.11	0.73–23.14	0.109
Medial medulla 0 1+	63 10	86.3 13.7	2.7	0.48–15	0.258	1.98	0.24–16.5	0.527
Lateral medulla 0 1+	68 5	93.1 6.9	4.5	0.31–65.45	0.274	23.15	0.52–1,032.45	0.105
Cerebellar vermis 0 1+	69 4	94.5 5.5	1.8	0.1–33.06	0.683	1.33	0.03–53.23	0.881
LL cerebellar hemisphere L 0 1+	47 26	64.4 35.6	2	0.54–7.37	0.296	2.46	0.51–11.88	0.262
LL cerebellar hemisphere R 0 1+	45 28	61.6 38.4	0.97	0.26–3.7	0.966	1.54	0.34–7	0.577

Each row represents the result of interest of two multivariable models (for depression and for anxiety), adjusted for age, sex, EDSS score, disease-modifying therapy, SDMT score, previous engagement with psychology/psychiatry, use of psychotropic medication, and brain volume

Unlike in the models of depression, larger brain volume was consistently associated with a higher risk of anxiety in all models (OR 1.35–1.41, *p*=0.017–0.032) (Table 4).

## Discussion

In this study, performed among 73 patients with multiple sclerosis in a cognitive neuroimmunology clinic at a single neuroimmunology centre, hyperintense T2 lesions involving raphe nuclei and locus coeruleus were associated with depression. We did not find evidence of an association between these structural changes and anxiety.

Anxiety tended to be more prevalent among women with MS than in men. This is consistent with results of a recent study, which reported a 1.78-times higher prevalence of anxiety in females with MS compared to males [26].

Patients with anxiety tended to show lower performance on SDMT than people without anxiety. The SDMT is a sensitive metric of neurocognitive function in MS measuring information processing speed [27]. Anxiety might interfere with brain networks involved in attention and cognition, thus resulting in poorer performance in tests of processing speed [28]. Our observation is in keeping with results of prior research in which anxiety detected with State-Trait Anxiety Inventory correlated with lower SDMT scores [29, 30]. Of note, our study was not sufficiently powered to replicate the well-known association between depression and low SDMT scores [3, 5, 8].

In this study, we chose LC as the most prominent noradrenergic nucleus and RN as a prominent representative of the serotoninergic network within the CNS. Our findings suggest that depression in MS is associated with structural changes within both noradrenergic and serotoninergic networks of the brainstem. This is further upheld by the observation that demyelinating lesions within the medial midbrain and medial pons conferred a higher risk of depression. On the other hand, the lack of association between depression and cerebellar lobule VIIA involvement suggests that our observations are specific to the brainstem structures. The functional topography of posterior non-motor cerebellum is complex and yet to be thoroughly defined. Cerebellar lobule VIIA appears to be involved in cognitive and affective processing through cerebellar contributions to the cognitive control network and the default mode network [31]. Its role in affective processing seems to be distinct from that of the serotoninergic and noradrenergic networks of the brainstem. Our findings imply that depression in MS is not fully attributable to the same pathogenetic mechanisms as primary depressive disorder but is likely secondary to structural changes triggered by demyelination. The findings also suggest depression in MS cannot be fully explained as a purely behavioural phenomenon.

The role of disrupted serotoninergic and noradrenergic brainstem networks in the pathogenesis of depression may extend beyond MS. In patients with Parkinson’s disease experiencing depression, reduced cellularity in RN, LC, and the ventral tegmental nuclei was reported [5]. This corresponded to a changed radiological appearance of the midline brainstem structures [32]. Similarly, ischaemic stroke involving RN was associated with significantly higher screening scores for depressive disorder [33]. Serotoninergic and noradrenergic nuclei of the brainstem project to the basal limbic system, and the temporal, frontal, and parietal areas of the networks involved in the control of mood and affect. Hyperintense T2 lesions involving these cortical areas have been more commonly found in patients with MS suffering from depression than those not depressed [5]. These findings suggest some of the neuroanatomical features of depression may be similar across diagnoses, while other pathophysiological processes may differ across conditions with differing aetiopathophysiology.

We did not find convincing evidence of associations between demyelinating lesions within RN or LC and anxiety. Studies in rodents showed that LC cellular loss resulted in increased anxiety [34, 35]. However, evidence of a similar association in humans is lacking. The higher risk of anxiety identified among patients with larger normalised brain volume may indicate that neuroanatomical changes in anxiety represent an epiphenomenon of behavioural mechanisms that may drive anxiety in a chronic neurological condition with uncertain prognosis such as MS.

Some limitations of the present study should be considered. Firstly, we relied on a relatively small sample of subjects. This could have precluded us from identifying subtle/minor associations of the topography of T2 lesions with anxiety or depression. Secondly, we did not gather data on other aspects possibly influencing the presentation of depressive and/or anxious symptomatology, especially the lack of support, stress levels, coping strategies, and the social and family history. Thirdly, the exact temporal relationships between MS and depression or anxiety were not established, allowing the possibility that some patients have been suffering from depression and/or anxiety already before the diagnosis of MS. We have attempted to mitigate the effect of the pre-existing mood disorders by adjusting the analyses for the use of psychotropic medications. For the assessment of the symptoms of depression and anxiety, we used SPECTRA Indices of Psychopathology, which has been validated for use in clinical practice, but, in isolation, it does not allow to establish the diagnosis of a mood disorder. This study only evaluated the regions of interest directly affected by T2-FLAIR lesions, but did not study their impact on pathways between clinically eloquent areas. A lesion within a certain brain structure can have variable effects on its supra- and infratentorial connective networks, implying an additional variability on the possibility of finding (or not) an association between a lesion site and a specific symptom. The locus coeruleus, for example, is an extremely complex anatomical structure with multiple projections contributing to a variety of systems. It has been observed that its neurones are clustered according to an output-specific topography identifiable on the dorso-ventral and anterior–posterior axes, meaning a lesion within LC can contribute to different clinical manifestations depending on the subpopulation of affected neurones and the pathway involved [36]. We were not able to apply fine discrimination of LC lesion localisation, which might explain the lack of significant association between lesioned LC and symptoms of anxiety. Finally, our study design allowed for analysis of associations, which should not be interpreted as causal.

The aetiology of mood disorders in MS is multifactorial. This study suggests that at least some of the mood disorders in MS are driven also by structural changes within the brain triggered by the autoimmune processes. This expands our understanding of depression in MS from the concept of reactive behavioural phenomenon to a condition with neuroanatomical predisposition that is specific to MS. Demyelinating lesions within the noradrenergic and serotoninergic networks of the brainstem can be identified with the presently available MRI techniques. While our findings suggest an association between these lesions and depression, their ability to predict susceptibility to depression in MS patients with no psychiatric history warrants further research. MRI may potentially serve as a screening tool for individual risk of depression in people with MS. Longitudinal studies tracking lesion development and mood changes over time are required to validate these findings. If the damage within the noradrenergic and serotoninergic networks proves to be a causal contributor to depression in MS, one could hypothesise that preventing formation of lesions within these locations with high-efficacy DMTs may be in part protective against future development of depression.

## Data Availability

The data that support the findings of this study are available from the corresponding author on *Figshare*: Giofre, Federica (2025). The topography of infratentorial lesions in depression and anxiety in multiple sclerosis. figshare. Dataset. https://doi.org/10.6084/m9.figshare.28381595.v1
